# The persistence of child and adolescence mental healthcare: results from registry data

**DOI:** 10.1186/s12913-020-05962-4

**Published:** 2020-12-02

**Authors:** Hermien H. Dijk, Roel D. Freriks, Rob J.M. Alessie, Jochen O. Mierau

**Affiliations:** 1 Department of Economics, Econometrics & Finance, University of Groningen, Nettelbosje 2, Groningen, 9747 AE The Netherlands; 2 Aletta Jacobs School of Public Health, University of Groningen, Landleven 1, Groningen, 9747 AD The Netherlands

**Keywords:** Psychiatric, Healthcare, Register Data, Panel data models

## Abstract

**Background:**

Previous studies on the persistence of child and adolescent mental healthcare do not consider the role of time-invariant individual characteristics. Estimating persistence of healthcare using standard linear models yields biased estimates due to unobserved heterogeneity and the autoregressive structure of the model. This study provides estimates of the persistence of child and adolescent mental healthcare taking these statistical issues into account.

**Methods:**

We use registry data of more than 80,000 Dutch children and adolescents between 2000 and 2012 from the Psychiatric Case Registry Northern Netherlands (PCR-NN). In order to account for autocorrelation due to the presence of a lagged dependent variable and to distinguish between persistence caused by time-invariant individual characteristics and a direct care effect we use difference GMM-IV estimation. In further analyses we assess the robustness of our results to policy reforms, different definitions of care and diagnosis decomposition.

**Results:**

All estimation results for the direct care effect (true state-dependence) show a positive coefficient smaller than unity with a main effect of 0.215 (*p*<0.01), which indicates that the process is stable. Persistence of care is found to be 0.065 (*p*<0.05) higher for females. Additionally, the majority of persistence of care appears to be associated with time-invariant characteristics. Further analyses indicate that (1) results are robust to different definitions of care and (2) persistence of care does not differ significantly across subgroups.

**Conclusions:**

The results indicate that the majority of mental healthcare persistence for children and adolescents is due to time-invariant individuals characteristics. Additionally, we find that in the absence of further shocks a sudden increase of 10 care contacts in the present year is associated with an average of less than 3 additional care contacts at some point in the future. This result provides essential information about the necessity of budget increases for future years in the case of exogenous increases in healthcare use.

## Background

The World Health Organization has categorised mental health problems as among the most disabling clinical diagnoses in the world [1]. Around 20% of the working age population in OECD countries are currently suffering from a mental disorder and the lifetime prevalence is even twice as high [2]. These disorders often originate from childhood [3, 4] and have long-lasting effects throughout the lifespan due to worse health and educational outcomes [5–8].

Since mental health problems appear to be highly persistent [3], it is important to understand whether child and adolescent mental healthcare is also persistent. If, in a certain year, there is an increase in the amount of mental healthcare required, knowledge on the persistence of that care provides information about the necessity of budget increases for future years. Consequently, understanding the persistence of care is also an important component of cost-effectiveness research, as it allows for a more accurate prediction of child and adolescent mental healthcare costs.

In addition, knowledge on the nature of the persistence of care in children and adolescents provides insights about the effectiveness of budget increases to reduce future healthcare use. If the persistence of care is largely the result of children’s time-invariant underlying characteristics, such as genetic predisposition [9], children currently in care are likely to receive care for many years to come, which, assuming the reception of care is strongly related to mental health states, suggests that care is mostly targeted at alleviating and managing symptoms but that it does not have long-term effects. In that case, broad budget increases in mental healthcare are unlikely to yield future reductions in required care, unless they alter the nature of the care provided.

If the role of individuals time-invariant characteristics is small, either mental health problems in themselves dissipate over time, care appears to have long-term effects, or the mechanism at work consists of a combination of both. We will refer to persistence that is not caused by time-invariant individual characteristics as the direct care effect of persistence (true state-dependence).

Only few studies have focused on the persistence of child and adolescent mental healthcare. Farmer et al. [10] and Shenkman et al. [11], find presence of persistence in child (mental) healthcare in the US, but do not consider the role of time-invariant individual characteristics in this persistence. Several studies on the persistence of child and adolescent mental health problems found that most of the persistence is likely to be due to time-invariant individual characteristics [12–14].

One might assume that this time-invariant persistence in mental health translates to time-invariant persistence of mental healthcare. However, not all individuals with mental health problems will automatically receive mental healthcare [15]. Additionally, studies on the persistence of all healthcare expenditures of elderly US citizens generally find that for these individuals, time-invariant individual characteristics appear to play a relatively small role in overall persistence of care [16, 17]. Hence, the mechanism underlying the persistence of child and adolescent mental healthcare remains unclear.

Therefore, this study investigates the nature of the persistence of child and adolescent mental healthcare by distinguishing between persistence due to time-invariant individual characteristics and the direct care effect. We do so using Dutch registry data of secondary psychiatric care of more than 80,000 children and adolescents in the Northern Netherlands, who received care between 2000 and 2012. The use of such a unique registry dataset results in a large representative sample of individuals in care in the Northern Netherlands. Furthermore, it circumvents reporting bias that might be present in survey self-reports of healthcare use [18]. Hence, this allows us to obtain estimates of persistence in daily practice, which enhances the generalizability of the results. Additionally, during the period of observation, three major reforms took place of which we analyse the effects.

## Methods

### Data

We use a unique registry dataset from the Psychiatric Case Registry Northern Netherlands (PCR-NN), which is a large longitudinal record of care contacts at the largest psychiatric institutions in the Northern Netherlands between 2000 and 2012. The PCR-NN contains year of birth, sex and diagnoses of the individuals in care, as well as entries denoting each care contact an individual received, which contained information on the date of the care contacts and the type of care.

As soon as individuals had their first appointment, or received their first diagnosis, at one of the institutions they entered the PCR-NN. Each separate appointment or diagnosis is a new entry in the dataset. An individual might not be observed in the original sample at a particular point of time for several reasons: (1) the individual did not receive secondary psychiatric care; (2) the individual did receive secondary psychiatric care, but not at a reporting institution; (3) the individual is deceased. This third possibility can be ruled out if at a later point that individual reappears in the set. Additionally, mortality in the Netherlands for the age group 5-25 was continuously below 0.03% for all years 2000–2012 [19, 20]. While the mortality rates for the individuals in our sample may be higher than those of the general population, they are unlikely to be so to a problematic degree as the direct mortality for mental illnesses is generally low [1, 21]. Furthermore, as previously mentioned, the institutions in the dataset accounted for most of the secondary psychiatric care provided in the Northern Netherlands. Consequently, it was assumed that individuals receive no secondary psychiatric care when they are not observed. With these assumptions we transformed the PCR-NN into a panel dataset with time intervals of one year.

The original sample of individuals aged 4 to 23 contains 5,975,096 observations of care contacts and diagnoses corresponding to 106,523 individuals. This sample was restricted to 5,083,812 care contacts and diagnoses from 93,786 individuals for Ordinary Least Squares (OLS) estimation, as a few of these care contacts were logged before January 2000 and estimation of persistence automatically excludes individuals with only one available time period. This data was transformed so that observations represented care contacts per year, leading to 485,072 observations from 93,786 individuals. Furthermore, identification of the direct care effect requires the availability of at least three consecutive time periods per individual. Consequently, the final sample for difference GMM-IV estimation contains a total of 391,286 care contacts per year from 81,525 individuals. Descriptive statistics of the OLS and difference GMM-IV samples are provided in Table 1.

**Table 1:** Descriptive statistics

	Mean	Standard deviation	Minimum	Maximum
OLS sample (N= 93,786)				
Year of birth	1992.30	5.39	1978	2007
Age	15.74	4.75	5	23
Female	0.41			
Care contacts per year per individual	8.36	36.57	0	764
Difference GMM-IV sample (N = 81,525)				
Year of birth	1992.27	5.08	1979	2006
Age	16.14	4.50	6	23
Female	0.40			
Care contacts per year per individual	7.09	34.55	0	764

### Estimation

We assume that the persistence of care can be described as

1 $$\begin{array}{@{}rcl@{}} Care_{i,t} &=& \beta_{1} Care_{i,t-1} + \boldsymbol{\beta}_{2} \boldsymbol{X}_{i,t} + c_{i} + \varepsilon_{i,t}, \end{array} $$

where *Care*_*i*,*t*_ and *Care*_*i*,*t*−1_ denote the number of care contacts individual *i* receives in year *t* and *t*−1, respectively, *c*_*i*_ captures unobserved time-invariant individual characteristics and ***X***_*i*,*t*_ is a vector of strictly exogenous control variables containing age and year dummies, *ε*_*i*,*t*_ denotes the error term and *β*_1_ is the parameter of interest, aimed to capture the direct care effect.

Equation 1 could be estimated using OLS if time-invariant individual characteristics, *c*_*i*_, are left out of the model. However, this estimation would yield inconsistent estimates of *β*_1_ and ***β***_2_ because *Care*_*i*,*t*−1_ is correlated with the unobserved time-invariant characteristics *c*_*i*_. To account for these time-invariant characteristics, we could estimate Eq. 1 using first differencing, effectively estimating:

2 $$\begin{array}{@{}rcl@{}} \Delta Care_{i,t} &=& \beta_{1} \Delta Care_{i,t-1} + \boldsymbol{\beta}_{2} \Delta \boldsymbol{X}_{i,t} +\Delta \varepsilon_{i,t}, \end{array} $$

where *ΔCare*_*i*,*t*_=*Care*_*i*,*t*_−*Care*_*i*,*t*−1_, *Δ****X***_*i*,*t*_=***X***_*i*,*t*_−***X***_*i*,*t*−1_ and *Δε*_*i*,*t*_=*ε*_*i*,*t*_−*ε*_*i*,*t*−1_.

Note that the right-hand side variable *ΔCare*_*i*,*t*−1_ is correlated with the error term *Δε*_*i*,*t*_ so that OLS estimation of Eq. 2 will yield inconsistent estimates. Additionally, first differencing introduces another source of autocorrelation since *Δε*_*i*,*t*_ and *Δε*_*i*,*t*−1_ both depend on *ε*_*i*,*t*−1_ [22]. To address these problems, we follow the suggestion of Arellano and Bond [23] and estimate Eq. 2 with Generalized Method of Moments with Instrumental Variables (GMM-IV) using past levels of care as instruments for *ΔCare*_*i*,*t*−1_. As excluded instrument, we use the first available lag of *Care*_*i*,*t*_ that does not cause the error term of the first stage to be correlated with *ε*_*i*,*t*_ [23] at a 10 percent significance level^1^. We only use a single lag to prevent problems due to too many, or weak, instruments [24].

Since prevalence rates for certain disorders can differ strongly by sex [25], persistence might also differ by sex. To test this, we perform the estimation separately for males and females. Additionally, we perform a number of sensitivity and robustness analyses. Firstly, we analyse how three different healthcare reforms might have changed the persistence of care over the period of observation. We test for a structural break in the persistence of care due to th Dutch healthcare reform in 2006 and the introduction of Diagnostic Treatment Combinations (DTCs) in 2008. We also assess how results change when we exclude the year 2012 from our analyses, when copayments were introduced for individuals aged 18 plus.

We also test whether our estimations are robust to different definitions of care. First, we re-estimate the model using cost estimates of care instead care contacts, after which we do the same using number of days per year an individual received care instead of care contacts. Additionally, as smaller time intervals might be of interest to policy makers, we vary the time unit of measurement by re-estimating the model again with number of care contacts per quarter - instead of number of care contacts per year - as our variable of interest.

Since persistence might vary by disorder, we perform separate estimations for individuals with a diagnosis of Attention-Deficit/Hyperactivity Disorders (ADHD), Pervasive Developmental Disorders (PDD), anxiety, and Episodic Mood Disorders (EMD) and any of their subtypes. Lastly, we estimate the persistence of care for the 5% highest care users in 2000, to investigate whether persistence differs based on an individuals’ position in the distribution of care contacts. All estimations are performed using Stata 15, the GMM-IV estimations are performed using the command xtabond2 [26].

## Results

### Main findings

Table 2 shows the estimates for *β*_1_ of Eq. 1,^2^. According to the Cumby-Huizinga test [28], there is evidence of autocorrelation of the error term in Eq. 2 both with its first and second lagged value (*p*<0.01 for both tests)(i.e., *Δε*_*i*,*t*_ is correlated to *Δε*_*i*,*t*−1_ and *Δε*_*i*,*t*−2_), suggesting that the model from Eq. 1 suffers from a MA(1) process of the residual term (*p*<0.01). In other words, *ε*_*i*,*t*_ appears to be correlated with *ε*_*i*,*t*−1_, but not with *ε*_*i*,*t*−2_. This autocorrelation process is likely the result of the inclusion of a lagged dependent variable. As a result, *Care*_*i*,*t*−3_ is the first valid instrument.

**Table 2:** Estimation results

	(1)	(2)	(2)
Care contacts	OLS	FE	d.GMM-IV
Care contacts (-1)	0.539***	0.189***	0.215***
	(0.0064)	(0.0016)	(0.0156)
Age dummies	YES	YES	YES
Year dummies	YES	YES	YES
Observations	485,072	485,072	391,286
R-squared	0.268	0.211	
Number of ID	93,786	93,786	81,525

d.GMM-IV: difference GMM-IV. YES: included in the estimation, NO: excluded from estimation. Robust standard errors in parentheses. Inference: *** *p* <0.01, ** *p* <0.05, * *p* <0.1. Cumby-Huizinga [28] autocorrelation test results yielded *p*-values of 0.000 {AR(1)}; 0.000 {AR(2)}; 0.215 {AR(3)}; 0.977 {AR(4)}

To maximize efficiency in availability of lags of *Care*_*i*_,*t*−1, we follow Arellano and Bond [23] and replace missing values for *Care*_*i*,*t*−3_ in the first stage equation by zeros. This will not decrease the validity of the results, but instead it increases efficiency by allowing for inclusion of observations with missing data in the IV in the second-stage regression [23, 29].

Since weak instruments might become a problem when using the third lag, we perform an F-test to determine the joint significance of the instruments for *ΔCare*_*i*,*t*−1_. We find an F-statistic of 1,746.95 (*p*<0.01) using cluster robust standard errors, which indicates that *Care*_*i*,*t*−3_ is a relevant instrument for *ΔCare*_*i*,*t*−1_^3^.

The difference GMM-IV estimate only captures the direct care effect and has a value of 0.215, which is smaller than unity, indicating that the process is stable. Hence, if children or adolescents experience a sudden increase in mental healthcare above a certain individual-specific base level of care in a certain year, they will receive an increased number of care contacts for the following years, but this effect will weaken over time so that eventually they will receive a base level of care again, as long as there are no further shocks. Hence, in the absence of further shocks, a sudden increase of 10 care contacts in the present year is associated with an average of less than 3 additional care contacts in the future above an individual’s long-term base-level.

In addition, the OLS estimate of Eq. 1 of 0.539 differs substantially from the difference GMM-IV estimate, suggesting that the majority of observed persistence is associated with time-invariant characteristics^4^. In other words, to a large extent, children currently in care appear to receive care for years^5^. If we assume that the reception of care is strongly related to children’s mental health states, this finding of the large role of time-invariant characteristics in the persistence of care suggests that a substantial amount of care might not have long-term effects but might instead be targeted at alleviating and managing symptoms.

Since prevalence rates for certain disorders can differ strongly by sex [25], persistence might also differ by sex. To test this, we perform the estimation separately for males and females. Results can be found in Table 3. For 23 individuals, sex was unknown, hence these individuals are excluded from the estimation. Females have a higher persistence of care than males (0.247 and 0.181, respectively). Both the interaction between the sex dummy and the lagged dependent variable and the F-test for the joint significance of all other interactions with the sex dummies are statistically significant (*p*<0.05). This suggests that the persistence of care is statistically significantly different for males and females and that different models should be performed by sex. This difference in persistence might be the result of different prevalence rates across different diagnoses between males and females [30].

**Table 3:** Sex differences

Care conacts	Full sample	Males	Females
Care contacts (-1)	0.181***	0.181***	0.247***
	(0.0207)	(0.0207)	(0.0236)
Care contacts (-1) × female	0.065**		
	(0.0314)		
Age dummies	YES	YES	YES
Year dummies	YES	YES	YES
Interaction terms females	YES	NO	NO
Observations	391,177	235,835	155,342
Number of ID	81,502	46,149	35,353

YES: included in the estimation, NO: excluded from estimation. Robust standard errors in parentheses. Inference: *** *p* <0.01, ** *p* <0.05, * *p* <0.1

### Policy reforms, definitions of care and decomposition

#### Policy reforms

We first assess the effects on the persistence of care of several healthcare reforms that took place in the period 2000–2012, using structural breaks. We find that the Dutch healthcare reform of 2006 did not statistically significantly affect persistence of care (*p*>0.10), whereas the introduction of Diagnosis Treatment Combinations (DTCs) in 2008 appears to be associated with a weakly statistically significant increase in the persistence of care (*p*<0.10). However, when we perform a combined F-test for 2008 and 2006 of the interactions between the structural breaks and *ΔCare*_*i*,*t*−1_ we find no statistical significance (*p*>0.10). The introduction of copayments for individuals aged 18 plus in 2012 does not affect our results: when we perform the estimation with and without the observations from that year the estimates for the direct care effect do not differ statistically significantly (*p*>0.10). Results can be found in Table 4.

**Table 4:** The 2006, 2008 and 2012 healthcare reforms

Care contacts	2006	2008	2012
Care contacts (-1)	0.183***	0.186***	0.201***
	(0.0280)	(0.0215)	(0.0170)
Care contacts (-1) × reform	0.047	0.064*	
	(0.0366)	(0.0374)	
Age dummies	YES	YES	YES
Year dummies	YES	YES	YES
Structural breaks	YES	YES	NO
Observations	391,286	391,286	332,907
Number of ID	81,525	81,525	74,259

YES: included in the estimation, NO: excluded from estimation. Robust standard errors in parentheses. Inference: *** *p* <0.01, ** *p* <0.05, * *p* <0.1

#### Definitions of care

Second, we test whether our estimations are robust to different definitions of care. First, we re-estimate the model using cost estimates of care instead care contacts, after which we do the same using number of days per year an individual received care instead of care contacts. Both results are extremely similar to our initial estimate, indicating that our initial results are robust to different definitions of care. We also vary the time unit of measurement by re-estimating the model again with number of care contacts per quarter - instead of number of care contacts per year - as our variable of interest. The results of this estimation show a coefficient for the direct care effect of persistence of 0.627 (*p*<0.01)^6^. This would indicate that, in the absence of further shocks, a sudden increase of 10 care contacts in the present quarter is associated with less than 17 additional care contacts at some point in the future. Results can be found in Table 5.

**Table 5:** Definitions of care

Care	Number of care days	Cost analysis	Care contacts per quarter
Care (-1)	0.224***	0.231***	0.627***
	(0.0147)	(0.0180)	(0.006)
Age dummies	YES	YES	YES
Year dummies	YES	YES	YES
Observations	391,286	391,286	2,009,510
R-squared			
Number of ID	81,525	81,525	100,515

YES: included in the estimation, NO: excluded from estimation. Robust standard errors in parentheses. Inference: *** *p* <0.01, ** *p* <0.05, * *p* <0.1

#### Diagnosis decomposition and highest care users

We also perform separate estimations for individuals with a diagnosis of Attention-Deficit/Hyperactivity Disorders (ADHD), Pervasive Developmental Disorders (PDD), anxiety, and Episodic Mood Disorders (EMD) and any of their subtypes. We find no statistically significant differences in the direct care effect between the different diagnosis groups (*p*>0.10, both for each diagnosis group independently and a combined F-test). When we estimate the direct care effect of persistence for the highest care users in 2000, we do find a higher of the direct care effect for these individuals, albeit not statistically significantly so (*p*>0.10). This lack of statistical significance is likely due to the relatively small number of individuals that were identified as highest care users. Results can be found in Table 6.

**Table 6:** Diagnosis groups and highest care-users

Care contacts	(1)	(2)	(3)	(4)	(5)
	ADHD	Anxiety	EMD	PDD	Highest care-users
Care contacts (-1)	0.181***	0.183***	0.220***	0.182***	0.364***
	(0.0282)	(0.0334)	(0.0438)	(0.0255)	(0.0966)
Age dummies	YES	YES	YES	YES	YES
Year dummies	YES	YES	YES	YES	YES
Observations	100,609	43,919	17,951	82,783	2,113
Number of ID	19,666	10,175	4,311	14,870	354

YES: included in the estimation, NO: excluded from estimation. Robust standard errors in parentheses. Inference: *** *p* <0.01, ** *p* <0.05, * *p* <0.1

## Discussion

In this paper we estimated a coefficient of the year-to-year direct care effect of persistence of Dutch secondary psychiatric care of 0.215. In the different sensitivity analyses, this coefficient varied depending on sex and the duration over which care was measured. Results also seemed to indicate that persistence was higher for the highest care users, but lacked statistically significance due to a small sample size. Future research could investigate further how persistence of care differs among high and low care-users.

Comparison of the OLS and difference GMM-IV results indicate that a substantial part of persistence is due to time-invariant individuals characteristics. These results seem to be in line with previous studies on the persistence of child and adolescent mental health problems [12–14]. For example, Wichstrøm et al. [14] find coefficients of 2-year homotypic persistence that, depending on the disorder, lie between 24% and 56% of estimates of persistence that also include persistence due to time-invariant characteristics.

This study is the first that considers the distinction between persistence of mental healthcare due to time-invariant characteristics and the direct care effect, which provides important information about the nature of care for policy makers and future research. Nevertheless, this study has some limitations, which we will discuss here.

The PCR-NN tracks individuals across institutions in the Northern Netherlands. However, not all institutions are included in the set, and individuals might obtain care at institutions outside the Northern Netherlands or in primary care. Consequently, at some point individuals in the set might have received secondary psychiatric care at institutions outside the set. Since we assume that individuals that are not observed receive no care, the true persistence of care might be underestimated. However, as previously mentioned, the PCR-NN covers most secondary psychiatric care in the Northern-Netherlands. Consequently, this bias is likely to be small.

Additionally, while the PCR-NN contains observations on a large number of individuals between 2000–2012, it lacks information on individual characteristics aside from sex, age and diagnoses. As such, the current study is unable to investigate which time-invariant characteristics in particular are responsible for the persistence of care not explained by the direct care effect. Hence, this is a topic for further research. Literature showing a strong correlation between socioeconomic status and certain mental health problems [31], as well as the probability of receiving care [32, 33], might lightly suggests that there might be a link between socioeconomic status and time-invariant persistence of care.

Lastly, in this study we perform a number of robustness and sensitivity analyses. It should be noted that the multiplicity problem might arise: the more analyses there are performed, the higher the probability that one or more of the results are generated by random chance.

Additionally, our estimates on the persistence of care should not be conflated with the necessity for care. There might be large groups of individuals with mental health problems who have never been in care and are, therefore, not represented in our sample [15]. Hence, budgeting decisions based on our estimates should take important factors in the accessibility of care into account, especially since individuals who might require care but are somehow unable to access it might be among the most vulnerable among society.

## Conclusion

This study investigated the persistence of child and adolescent mental healthcare use between 2000 and 2012 with registry data of more than 80,000 Dutch children and adolescents. The results indicate that a substantial part of persistence is due to time-invariant individuals characteristics. Additionally, we find a coefficient for the direct care effect of 0.215 (*p*<0.01). Specifically, the main result implies that in the absence of further shocks a sudden increase of 10 care contacts in the present year is associated with an average of less than 3 additional care contacts at some point in the future. This result provides essential information about the necessity of budget increases for future years in the case of exogenous increases in healthcare use.

## Data Availability

The data in this study are available from the PCR-NN but restrictions apply to the availability of these data, which were used under general conditions for the current study, and are therefore not publicly available.

## Abbreviations

AR: Autoregressive
ADHD: Attention-deficit/hyperactivity disorder
DTC: Diagnosis treatment combination
EMD: Episodic mood disorders
GMM-IV: General method of moments with instrumental variables
MA: Moving average
PCR-NN: Psychiatric case registry Northern Netherlands
PDD: Pervasive developmental disorders
OLS: Ordinary least squares

## Footnotes

1
*C**a**r**e*_*i*,*t*−2_ of *C**a**r**e*_*i*,*t*_ will be a valid instrument as long as the error term *ε*_*i*,*t*_ (cf. Eq. 1) is serially uncorrelated. It then holds that *E*(*C**a**r**e*_*i*,*t*−2_*Δ**ε*_*i*,*t*_)=0. Note also that *Δ**ε*_*i*,*t*_ follows a MA(1) process (*c**o**v*(*Δ**ε*_*i*,*t*_,*Δ**ε*_*i*,*t*−1_)<0 and *c**o**v*(*Δ**ε*_*i*,*t*_,*Δ**ε*_*i*,*t*−*k*_)=0, *k*≥2) if *ε*_*i*,*t*_ is serially uncorrelated. We will carry out a Cumby-Huizinga test to select the lag to use as instrument. Firstly, we will check if the test is able to reject the hypothesis of lack of serial dependence of *Δ**ε*_*i*,*t*_ in Eq. 2 using *C**a**r**e*_*i*,*t*−2_ as excluded instrument for *Δ**C**a**r**e*_*i*,*t*−1_. Then we will run difference GMM-IV regressions with *C**a**r**e*_*i*,*t*−(*k*+1)_ as excluded instrument for *Δ**C**a**r**e*_*i*,*t*−1_, starting with *k*=2. We will use the same test iteratively, to check the validity of the following hypothesis: *Δ**ε*_*i*,*t*_ follows a *k* order MA process, MA(*k*) with *k*≥2. If the test results yields a rejection of the null hypothesis in favor of an AR(*k*+1) process, we will use *C**a**r**e*_*i*,*t*−(*k*+2)_ as excluded instrument for *Δ**C**a**r**e*_*i*,*t*−1_ (thus we will update the value of *k*, by adding 1). We will continue until a value *k*^∗^ is found for which the test is not rejected: $\phantom {\dot {i}\!}Care_{i,t-(k^{*}+1)}$ will be a valid instrument then.

2 The FE estimate, *β*_1_ in Eq. 2, functions as a first check, as the difference GMM-IV estimate should lie between the OLS and FE estimates [27]. The results demonstrate that this is the case and, consequently, that the difference GMM-IV is likely consistent.

3 We also extended the set of instruments by including interactions between year dummies and *C**a**r**e*_*i*,*t*−3_. The estimation results are barely affected by the inclusion of those extra instruments. Additionally, when we also include *C**a**r**e*_*i*,*t*−4_ up to *C**a**r**e*_*i*,*t*−10_ as excluded instruments, results do not change substantially: the direct care-effect ranges between 0.218 (*p*<0.01) and 0.230 (*p*<0.01), depending on the number of lags used as excluded instruments.

4 This result that the OLS estimate is more than double the d.GMM-IV estimate also holds for all further analyses, with the exception of the highest care users and quarterly persistence. OLS results for the further analyses are available upon request.

5 Since OLS estimation requires less available lags of *C**a**r**e*_*i*,*t*_ the sample differs slightly from the sample used for difference GMM-IV estimation. Consequently, we have also performed the same OLS estimation using the sample used for difference GMM-IV. This estimation resulted in a very similar coefficient of 0.522 (*p*<0.01).

6 Peforming OLS using number of care contacts per quarter results in an estimate of persistence of 0.778 (*p*<0.01)(results available upon request). Hence, the conclusion that a large share of the persistence of care is due to time-invariant individual characteristics is confirmed by this analysis, as this small quarterly difference between overall persistence of care and the direct care-effect of persistence will (given either the model in Eq. 1 or 2) result in a large yearly difference where the majority of yearly persistence can be explained by time-invariant individual characteristics.
